# A Deviation from the Orthodox Method of Treating Parturient Paresis and Its Etiology

**Published:** 1902-07

**Authors:** W. E. A. Wyman

**Affiliations:** Portland, Mich.


### THE JOURNAL

OF

### COMPARATIVE MEDICINE AND VETERINARY ARCHIVES.

VOL. XXIII. *JULY, 1902.* No. 7.

### A DEVIATION FROM THE ORTHODOX METHOD OF TREATING PARTURIENT PARESIS AND ITS ETIOLOGY.

BY W. E. A. WYMAN, V.S.,
PORTLAND, MICH.

SCHMIDT-COLDING, as any reading veterinarian knows, suggested the iodide of potassium water udder infusion in the treatment of parturient paresis. This treatment when properly executed is certainly reliable and reduces the rate of mortality to an astonishing degree. The writer, after an extensive experience with udder infusion, firmly believes that udder infusions are a condition *sine qua non* in the treatment of this disease. Schmidt-Colding claims that the iodide of potassium conquers the toxin supposed by him to be formed in the udder, laying the foundation of an auto-intoxication which he claims this disease represents. With all due respect to Dr. Schmidt-Colding, the writer does not at all agree with his propositions. Since eighteen months the writer has used no iodide of potassium at all, but either simply boiled water or boiled water and sodium chloride—that is, a normal saline solution. Either one of these is just as prompt in its action as the iodide of potassium water solution; the amount the writer uses is governed entirely by the size of the udder, it being his intention to distend the udder for reasons subsequently given. This point alone settles the necessity of employing iodide of potassium, and explodes Dr. Schmidt's theory that the iodine set free from the iodide of potassium should be the curative agent. The writer does not believe in an auto-intoxication due to products of decomposition arising in the udder to be the cause of parturient paresis, but believes that the cause of the disease is to be found in a sudden undue accumulation of nutritive elements in the maternal body as soon as the umbilical cord tears. As long as the fœtus is supported by the mother the distribution of nutritive elements is balanced or reasonably balanced, which is done away with as soon as the calf is born. If at this moment the udder secretes fully, this excess of nutritive elements is excreted via the udder and the animal remains well, but should the udder do its duty but partially this excess of nutritive elements is retained in the body, and an auto-intoxication can take place. The udder infusion most likely either takes up the poison, or by its presence excites the parenchyma of the gland into renewed activity, thus draining the excess of nutritive elements stored up in the body and doing away with the foundation of an auto-intoxication. On the other hand, instead of exciting the udder into activity, the infusion may set the gland at rest by the pressure which it exerts upon the parenchyma. Should this be correct, what explanation can be offered as regards the curative action of the saline infusion? It is probably not to be denied that at this time an excess of blood invades the udder, as a result of which anæmia of the brain and a comatose, or at least depressed, state of the senses follow. This should explain both ante- and post-partum cases.

By distending the udder with the saline infusion its activity is possibly arrested, and thus a chance given for the equalization of the disturbed circulation, thus a removal of the anæmic state of the brain and a return to health. The first theory advanced by the writer appears the stronger one, while the latter one undoubtedly has points which recommend themselves. Will our reading veterinarians ventilate their views and help us fix our creed in the etiology of this interesting disease? The longer the writer treats parturient paresis the more does he become convinced that we occasionally imagine we have a case of parturient paresis, but in reality are dealing with a disease simulating parturient paresis closely; we are dealing with a parturient paresis-like disease; in other words, have before us a case of acute puerperal septicæmia. Does not the fact that these intramammary infusions sometimes utterly fail in recent cases without marked organic complications on post-mortem hint at that?

